# Endothelinergic Contractile Hyperreactivity in Rat Contralateral Carotid to Balloon Injury: Integrated Role for ET_B_ Receptors and Superoxide Anion

**DOI:** 10.1155/2017/3137580

**Published:** 2017-09-14

**Authors:** Larissa Pernomian, Lilian R. Gimenes, Mayara S. Gomes, Bruno N. do Vale, Cristina R. B. Cardoso, Ana M. de Oliveira, Josimar D. Moreira

**Affiliations:** ^1^Department of Biosciences Applied to Pharmacy, Faculty of Pharmaceutical Sciences of Ribeirão Preto, University of São Paulo, Ribeirão Preto, SP, Brazil; ^2^Department of Pharmacology, Faculty of Medicine of Ribeirão Preto, University of São Paulo, SP, Brazil; ^3^Department of Physics and Chemistry, Faculty of Pharmaceutical Sciences of Ribeirão Preto, University of São Paulo, Ribeirão Preto, SP, Brazil; ^4^Department of Pharmacy, University Center UnirG, Gurupi, TO, Brazil; ^5^Department of Clinical and Toxicological Analysis, Faculty of Pharmacy, Federal University of Minas Gerais, Belo Horizonte, MG, Brazil

## Abstract

Temporal consequences of neurocompensation to balloon injury on endothelinergic functionality in rat contralateral carotid were evaluated. Rats underwent balloon injury in left carotid and were treated with CP-96345 (NK_1_ antagonist). Concentration-response curves for endothelin-1 were obtained in contralateral (right) carotid at 2, 8, 16, 30, or 45 days after surgery in the absence or presence of BQ-123 (ET_A_ antagonist), BQ-788 (ET_B_ antagonist), or Tempol (superoxide-dismutase mimic). Endothelin-1-induced calcium mobilization was evaluated in functional assays carried out with BQ-123, BQ-788, or Tempol. Endothelin-1-induced NADPH oxidase-driven superoxide generation was measured by lucigenin chemiluminescence assays performed with BQ-123 or BQ-788. Endothelin-1-induced contraction was increased in contralateral carotid from the sixteenth day after surgery. This response was restored in CP-96345-treated rats. Endothelium removal or BQ-123 did not change endothelin-1-induced contraction in contralateral carotid. This response was restored by BQ-788 or Tempol. Contralateral carotid exhibited an increased endothelin-1-induced calcium mobilization, which was restored by BQ-788 or Tempol. Contralateral carotid exhibited an increased endothelin-1-induced lucigenin chemiluminescence, which was restored by BQ-788. We conclude that the NK_1_-mediated neurocompensatory response to balloon injury elicits a contractile hyperreactivity to endothelin-1 in rat contralateral carotid by enhancing the muscular ET_B_-mediated NADPH oxidase-driven generation of superoxide, which activates calcium channels.

## 1. Introduction

Vascular remodeling is a hallmark of many vascular disorders including atherosclerosis [1, 2]. Carotid occlusive disease is a specific kind of atherosclerosis that significantly contributes to cerebrovascular accidents [3]. In turn, stroke represents one of the main leading causes of the mortality assigned to cardiovascular diseases, which account for 7.6 million of deaths annually [4].

Balloon angioplasty is the most common intervention to restore blood flow upon arterial obstruction by atherosclerotic plaques [5–7]. However, therapeutic efficacy of balloon angioplasty is limited by postoperative complications mainly resultant from restenosis, which markedly narrows ipsilateral (injured) artery lumen and reduces local blood flow [8, 9].

Pathophysiological mechanisms underlying restenosis have been effectively studied by the rat carotid balloon injury model, which triggers neointimal formation in close similarity to human remodeling [1, 9, 10]. Recent findings obtained with this model have strongly supported that postangioplasty complications also comprise harmful effects at the noninjured (contralateral) carotid. These effects acutely enhance *α*_1_-mediated adrenergic functionality (2 to 7 days after surgery) and chronically upregulate AT_1_-mediated angiotensinergic functionality (15 to 30 days after surgery) in contralateral carotid by reactive oxygen species- (ROS-) dependent mechanisms that impairs endothelial function and increases muscular calcium (Ca^2+^) mobilization [5, 6, 11–16].

Contractile hyperreactivity of contralateral carotid makes it more sensitive to mechanical load pressure effects of increased vascular resistance conditions, which contributes to a further impairment of cerebral flow already reduced by the ipsilateral restenotic remodeling [5, 14, 16]. Thus, the elucidation of the mechanisms underlying the distant harmful effects of balloon angioplasty has become one of the main clinical interests for overcoming the postoperative complications that limit its therapeutic efficacy. The first findings regarding this matter showed that these effects result from a neurocompensatory response that increases the density of substance P- (SP-) and calcitonin gene-related peptide- (GPCR-) containing nerves at contralateral carotid as an immediate consequence of the damage of perivascular nerve density from ipsilateral carotid during catheter rubbing [11, 14, 16]. In turn, SP and CGRP acutely upregulate the expression of contractile factors underlying *α*_1_-adrenergic signaling such as cyclooxygenase-2 (COX-2) [17]. Overexpressed COX-2 yields high levels of superoxide anion (O_2_^−^), which induces endothelial dysfunction and the redox-mediated activation of muscular Ca^2+^ channels, leading to adrenergic contractile hyperreactivity of contralateral carotid [5, 6, 12, 13]. The overactivation of *α*_1_-adrenoceptors is an inhibitory mechanism of AT_1_-mediated signaling [18], which stays subregulated in contralateral carotid during the adrenergic hyperreactivity [5]. Interestingly, when local adrenergic contractile tone is recovered by the fifteenth day after surgery [5, 15], the adrenergic inhibitory mechanism of AT_1_-mediated signaling is blunted, which prompts the compensatory upregulation of local angiotensinergic functionality by a ROS-dependent endothelial dysfunction that makes contralateral carotid hyperreactive to angiotensin II (AngII) until the thirtieth day after surgery [5, 16]. Contralateral carotid tone is only restored when local endothelial function is recovered by the forty-fifth day after surgery, concomitantly to ipsilateral reendothelialization and sensorial repair [5, 11].

Other vasoactive systems than the adrenergic and angiotensinergic ones are important mediators of angioplasty late harmful effects: endothelin-1 (ET-1) can be pointed as one of the main chronic mediators of restenosis due to the wide expression of endothelin receptors in remodeled vasculature [19]. Interestingly, endothelinergic system is in a crosstalk with angiotensinergic system that makes AT_1_ activation upregulate the expression of ET-1, which finally mediates AngII redox and ionic effects [20]. Based on these findings, we hypothesized that neurocompensatory response to balloon injury enhances endothelinergic functionality in contralateral carotid, leading to local contractile hyperreactivity to ET-1 by a ROS-dependent mechanism involving Ca^2+^ mobilization upregulation. Thus, we aimed to investigate the temporal consequences of balloon angioplasty on endothelinergic functionality in rat contralateral carotid. Elucidating the mechanisms underlying distant harmful effects of balloon injury and their pathophysiological significance may contribute to the development of effective therapeutic approaches to prevent postangioplasty complications.

## 2. Materials and Methods

The present study was carried out with adult male Wistar rats provided by the Central Vivarium from the University of São Paulo (USP). Animals were kept in a 12 h light–12 h dark cycle at room temperature (22°C) and relative humidity of 60%. Free access to food and water was allowed to rats. The experimental protocols were performed in accordance with the Guide for the Care and Use of Laboratory Animals upon a prior approval granted by the Ethics Committee on Animal Use (CEUA) from the University of São Paulo (USP), Ribeirão Preto Campus, Brazil (grant number: 120/2007).

### 2.1. Balloon Catheter Injury

Unilateral balloon injury was carried out by introducing a 2F Fogarty balloon catheter in the left common carotid artery (ipsilateral vessel) from rats previously anaesthetized with ketamine (50 mg/kg, i.p.) and xylazine (50 mg/kg, i.p.). Rats with an average age of 75 days (400–450 g) were killed at 8, 16, 30, or 45 days after the surgery. Functional data obtained in carotid arteries from sham-operated rats were not different from those obtained in carotid arteries from intact (nonoperated) rats (see Supplementary Material available online at https://doi.org/10.1155/2017/3137580). Thus, we have used intact rats as the control group to avoid unnecessary animal suffering [5, 6, 12–16].

In order to confirm the involvement of SP as the mediator of neurocompensatory response to balloon injury on the functional changes in contralateral carotid, rats were chronically treated with the selective SP NK_1_ receptor antagonist CP-96345 (5 mg/kg/day i.p., divided into two daily doses of 2.5 mg/kg, that were administered every 12 h) for 45 days after the surgery. Age-matched intact control rats were also treated with CP-96345.

### 2.2. Histological Assays

Ipsilateral (left injured) and contralateral (right noninjured) carotid arteries were removed from operated rats. Control carotid arteries were removed from intact rats. Carotid segments were fixed with formalin (10%) for 24 h and embedded in paraffin. 4 *μ*m thick sections were stained with hematoxylin and eosin (HE) for morphological analysis in optic microscopy coupled to a digital camera (Coolpix 4500, Roper Scientific, Japan). The images were edited in the Adobe Photoshop CS3 software [21].

### 2.3. Functional Vascular Reactivity Assays

Isoflurane-anaesthetized rats were killed by aortic exsanguination for the remove of common carotid arteries. Carotid rings (4 mm) were placed in organ bath chambers (10 ml) for isolated organs containing 5.0 ml of Krebs-Henseleit bicarbonate buffer (composition in mmol/l: NaCl 118.4; KCl 4.7; CaCl_2_ 1.9; KH_2_PO_4_ 1.2; MgSO_4_·7H_2_O 1.2; NaHCO_3_ 25; glucose 11.6) gassed with carbogenic mixture (95% O_2_ and 5% CO_2_), kept at 37°C (pH 7.4), and underwent periodic checking [14, 22, 23]. Carotid rings were connected to isometric force transducers (Letica Scientific Instruments, Barcelona, Spain) and underwent a resting tension of 1.0 g, which was readjusted every 15 min throughout a 60 min lasting equilibration period. After stabilization, the viability of vessel rings was evaluated with potassium chloride (KCl, 90 mmol/l) or phenylephrine (PE, 0.1 *μ*mol/l). Endothelial integrity was assessed by the degree of relaxation induced by acetylcholine (ACh, 1.0 *μ*mol/l) over PE-induced precontraction [24]. In order to evaluate the modulation played by endothelium on carotid functionality, some experimental protocols were performed in endothelium-denude carotid rings. For these purposes, endothelium was mechanically removed by gently rubbing the intimal surface of carotid rings with a thin wire. The endothelium was considered removed if the relaxant response to ACh was abrogated [25].

In order to evaluate the endothelinergic contractile functionality, cumulative concentration-response curves for ET-1 (1.0 pmol/l–0.1 *μ*mol/l) were obtained in endothelium-intact (E+) or endothelium-denuded (E−) carotid rings pretreated or not with the selective ET type A receptor (ET_A_) antagonist BQ-123 (3.0 *μ*mol/l), the selective ET type B receptor (ET_B_) antagonist BQ-788 (3.0 *μ*mol/l) [26], or the superoxide-dismutase (SOD) mimic Tempol (1.0 mmol/l) combined or not with the hydrogen peroxide (H_2_O_2_) scavenger PEG-catalase (250 U/mL) [12, 27], added 30 min prior to ET-1. Also, we evaluated the endothelinergic relaxant functionality by obtaining the maximum relaxation induced by ET-1 (0.1 nmol/l) in PE (0.1 *μ*mol/l) precontracted E+ or E− carotid rings in the absence or presence of BQ-123 (3.0 *μ*mol/l) or BQ-788 (3.0 *μ*mol/l), added 30 min prior to ET-1 [26].

In order to assess intracellular Ca^2+^ mobilization induced by ET-1, the Krebs solution was replaced with a Ca^2+^-free solution and then the contraction was stimulated with ET-1 (0.1 mmol/l) in the absence or presence of BQ-123 (3.0 *μ*mol/l), BQ-788 (3.0 *μ*mol/l), or Tempol (1.0 mmol/l), added 30 min prior to ET-1 [28]. ET-1-induced extracellular Ca^2+^ mobilization protocol was carried out by depleting intracellular Ca^2+^ stores upon the stimulation of carotid rings with PE (0.1 *μ*mol/l) in Ca^2+^-free solution containing the Ca^2+^ chelator ethylene glycol-bis(aminoethyl ether)tetraacetic acid (EGTA, 10 *μ*mol/l) and the repeated rinse until there was no contractile response; then, carotid rings were stimulated with ET-1 (0.1 mmol/l) in the presence of a solution containing Ca^2+^ (1.9 mmol/l) [28].

In order to assess the depolarization-dependent contraction, cumulative concentration-response curves for KCl (10–120 mmol/l) were obtained in E+ carotid rings [29].

Analysis of concentration-response curves were fitted using the nonlinear interactive fitting program GraphPad Prism 5.0 (GraphPad Software Inc., San Diego, CA) [30]. The maximum contractile effect elicited by ET-1 or KCl (*E*max) was expressed in grams of force per milligram of dry tissue weight and determined from the concentration-response curves that were analyzed by computer-assisted nonlinear regression to fit the data [31–33].

### 2.4. Lucigenin Chemiluminescence Assays

In order to measure the basal and ET-1-induced NAD(P)H oxidase-driven generation of O_2_^−^, lucigenin chemiluminescence assays were performed in carotid homogenates. In brief, carotid rings were frozen at −80°C before equilibration in Krebs-Henseleit bicarbonate buffer (composition in mmol/l: NaCl 118.4; KCl 4.7; CaCl_2_ 1.9; KH_2_PO_4_ 1.2; MgSO_4_·7H_2_O 1.2; NaHCO_3_ 25; glucose 11.6,* p*H 7.4, 37°C) for 30 min. Frozen rings were macerated with a glass-to-glass homogenizer in phosphate buffer (EGTA 1 mmol/L + KH_2_PO_4_ 20 mmol/L + protease inhibitors,* p*H 7.4). NAD(P)H (0.1 mmol/L) was added to the suspension of homogenates 10% (w/v) (250 *μ*L of final volume) containing the sample (50 *μ*L), the assay buffer (KH_2_PO_4_ 50 mmol/L + EGTA 1 mmol/L + sucrose 150 mmol/L,* p*H 7.4), and lucigenin (5.0 *μ*mol/L). Luminescence was measured in a luminometer (Orion II Luminometer, Berthold Detection Systems) every 1.8 s for 3 min. After discounting buffer blank luminescence signal from sample luminescence signal, the final value was normalized by tissue protein mass (mg). NADPH oxidase-driven O_2_^−^ generation was expressed as relative light unities (RLU) per mg of protein. Protein concentrations were determined with the Bradford assay (BioRad) [14]. The role of ET_B_ receptors on ET-1-induced NAD(P)H oxidase-driven O_2_^−^ generation was determined in carotid homogenates pretreated with BQ-788 (3.0 *μ*mol/l), added 30 min before ET-1 (0.1 *μ*mol/L) stimulation, which was followed by immediate sample freezing [26].

### 2.5. Amplex Red Assays

In order to evaluate the putative ET-1-induced generation of O_2_^−^-derived H_2_O_2_, Amplex Red assays were carried out in carotid homogenates. Vascular rings were frozen in liquid nitrogen (−196°C), stored at −80°C, homogenized in Krebs-Henseleit bicarbonate buffer (composition in mmol/l: NaCl 118.4; KCl 4.7; CaCl_2_ 1.9; KH_2_PO_4_ 1.2; MgSO_4_·7H_2_O 1.2; NaHCO_3_ 25; glucose 11.6,* p*H 7.4, 37°C), and centrifuged at 14.770 ×g under refrigeration (each *n* comprised a pool of 4 arteries). The reagents from the Amplex Red hydrogen peroxide assay kit (Molecular Probes, Invitrogen, Carlsbad, CA-USA) were added in the supernatant from the homogenates according to the protocol provided by the manufacture. Hydrogen peroxide levels were determined by enzyme-linked immunosorbent assay (ELISA). Sample H_2_O_2_ levels were measured in samples stimulated or not with ET-1 (0.1 *μ*mol/L, before sample freezing) by using a standard solution of H_2_O_2_ incubated with the UltraRed working solution (100 *μ*mol/L) at 37°C as the standard curve on the same 96-well plate used for the supernatants. Fluorescence emission was detected on the Biotek Synergy HT plate reader at excitation of 530 nm and emission of 590 nm. H_2_O_2_ levels were expressed as absolute micromolar concentrations (*μ*mol/L) [14].

### 2.6. Data Analysis

Data were expressed as the mean ± SEM (standard error of the mean) and the differences between the mean values were assessed using the one-way analysis of variance (ANOVA) followed by the Bonferroni post hoc test. The significance level considered in all of the tests was 0.05 [34].

## 3. Results and Discussion

### 3.1. Results

#### 3.1.1. Histological Data

**Figure 1:**
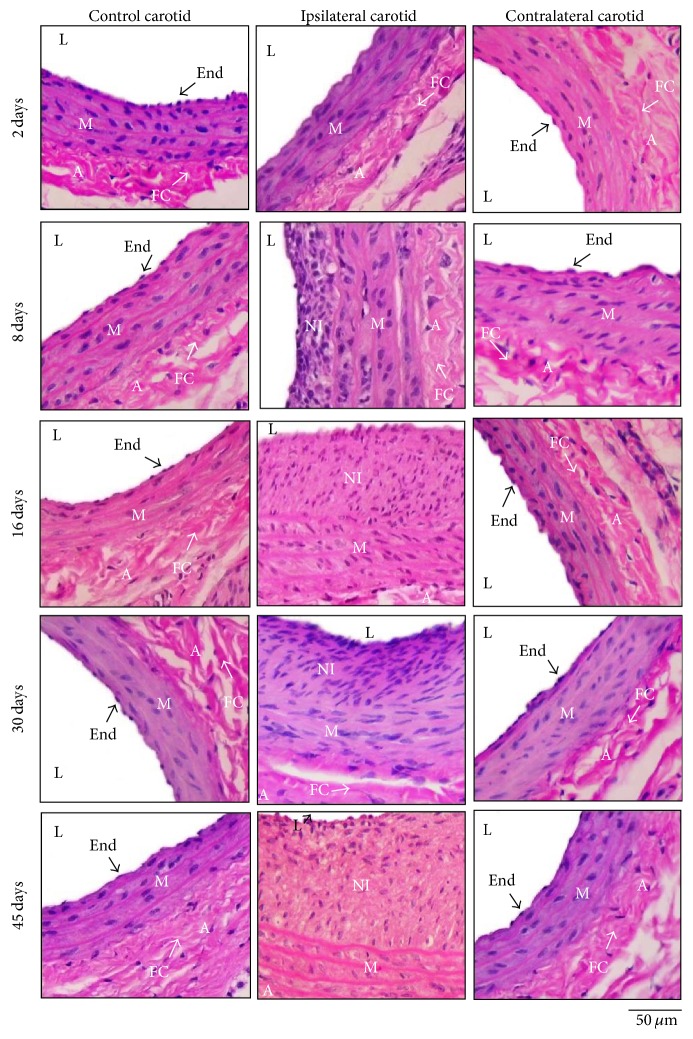
Representative histological images from ipsilateral or contralateral carotid arteries isolated from operated rats at 2, 8, 16, 30, or 45 days after the balloon catheter injury, compared to representative histological images from control carotid arteries isolated from age-matched intact rats. HE staining, 40x. L: lumen; End: endothelium; NI: neointima; M: media; A: adventitia; FC: collagen fibers.

Morphological analysis showed that rat contralateral carotid rings were not different from the respective age-matched rat control carotid rings. In turn, ipsilateral carotid rings exhibited balloon-elicited endothelium denudation, which triggered a gradual neointimal proliferation followed by irregular reendothelialization from the eighth day till the forty-fifth day after surgery (Figure 1).

#### 3.1.2. Functional Data

**Figure 2:**
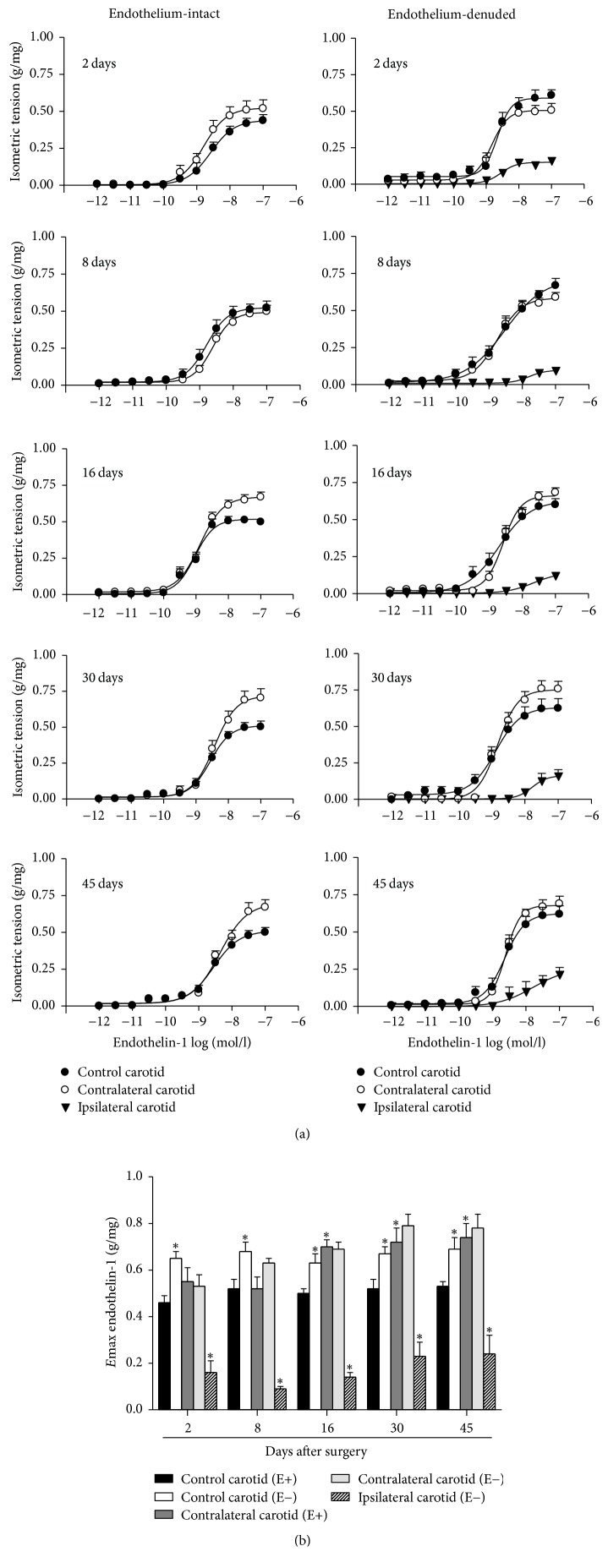
Temporal consequences of balloon injury on endothelinergic functionality in ipsilateral or contralateral carotid rings isolated from operated rats at 2, 8, 16, 30, or 45 days after the surgery. (a) Cumulative concentration-response curves for ET-1 in endothelium-intact (E+) or endothelium-denuded (E−) carotid rings. (b) *E*max values from ET-1-induced contraction in E+ or E− carotid rings. Significant difference (*P* < 0.05) from E+ control carotid rings from the respective age-matched intact rat (*∗*). One-way ANOVA; Bonferroni post hoc test.

**Figure 3:**
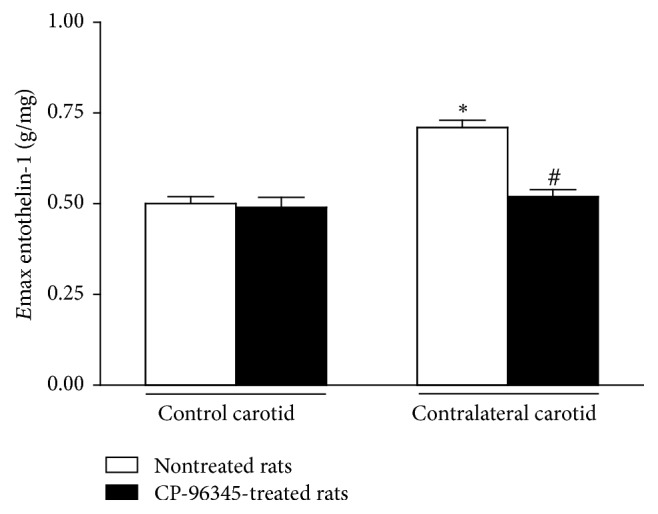
Effect of CP-96345 (SP NK_1_ receptors antagonist) on ET-1 *E*max values in endothelium-intact (E+) contralateral carotid rings isolated from operated rats at the sixteenth day after surgery and in E+ age-matched rat control carotid rings. Significant difference (*P* < 0.01) from E+ control carotid rings of nontreated rats (*∗*) or E+ contralateral carotid rings from CP-96345-treated rats (#). One-way ANOVA; Bonferroni post hoc test.

*(1) Endothelinergic Functionality*. Balloon injury reduced ET-1 *E*max values in ipsilateral carotid rings removed from operated rats at 2, 8, 16, 30, or 45 days after surgery in the same extent. ET-1 *E*max values were similarly increased in contralateral carotid rings from the sixteenth day till the forty-fifth day after surgery when compared to the respective control carotid rings isolated from age-matched intact rats. Endothelium removal increased ET-1 *E*max values in control carotid rings but did not change the endothelinergic contraction in contralateral carotid rings when compared to the respective endothelium-intact groups (Figure 2). CP-9634-treatment did not change ET-1 *E*max values in E+ control carotid rings (0.49 ± 0.028 g/mg, *n* = 9) but restored this response in E+ contralateral carotid rings removed from operated rats at the sixteenth day after surgery (0.52 ± 0.019 g/mg, *n* = 9) (one-way ANOVA; Bonferroni post hoc test, *P* < 0.01) (Figure 3).

**Figure 4:**
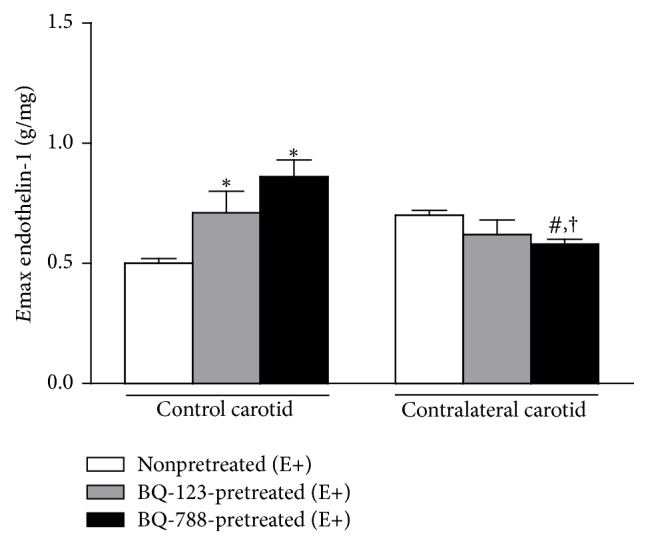
Effect of ET_A_ (BQ-123) or ET_B_ (BQ-788) antagonists on ET-1 *E*max values in endothelium-intact (E+) contralateral carotid rings isolated from operated rats at the sixteenth day after surgery and in E+ age-matched rat control carotid rings. Significant difference (*P* < 0.05) from E+ nonpretreated control carotid rings (*∗*), E+ nonpretreated contralateral carotid rings (#), or E+ control carotid rings pretreated with the respective antagonist (†). One-way ANOVA; Bonferroni post hoc test.

BQ-123 pretreatment increased ET-1 *E*max values in E+ control carotid rings but did not change this response in E+ contralateral carotid rings removed from operated rats at the sixteenth day after surgery. In turn, BQ-788 pretreatment increased ET-1 *E*max values in E+ control carotid rings but restored the endothelinergic contraction in E+ contralateral carotid rings removed from operated rats at the sixteenth day after surgery to the levels obtained in nonpretreated E+ control carotid rings from age-matched rats (Figure 4).

**Figure 5:**
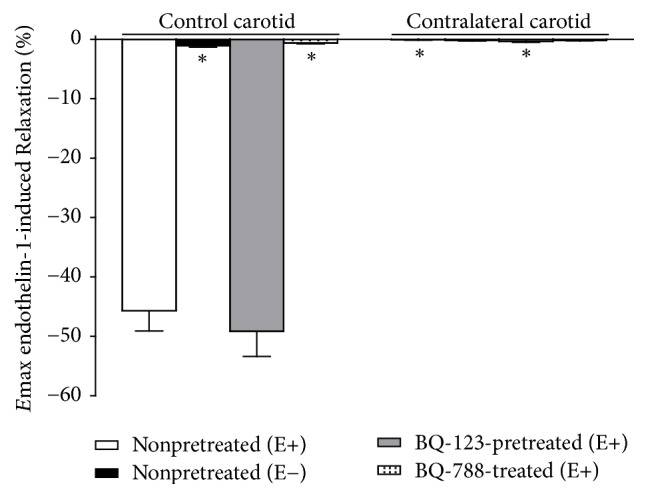
Effects of ET_A_ (BQ-123) or ET_B_ (BQ788) antagonists on endothelinergic relaxation *E*max values in contralateral carotid rings isolated from operated rats at the sixteenth day after surgery. Significant difference (*P* < 0.01) from the respective rat control carotid rings (*∗*). One-way ANOVA; Bonferroni post hoc test.

ET-1-induced relaxation over PE-precontracted E+ control carotid rings (*E*max = 45.71 ± 3.19%, *n* = 9) was blunted by endothelium removal (*E*max = 1.09 ± 0.24%, *n* = 9) or BQ-788 pretreatment (*E*max = 0.65 ± 0.12%, *n* = 9) but was not altered by BQ-123 pretreatment (*E*max = 49.16 ± 4.05%, *n* = 9). This response was completely absent in both PE-precontracted E− ipsilateral carotid rings (*E*max = 0.07 ± 0.09%, *n* = 9) or E+ contralateral carotid rings (*E*max = 0.23 ± 0.14%, *n* = 9) removed from operated rats at the sixteenth day after surgery. Neither endothelium removal (*E*max = 0.18 ± 0.12%, *n* = 9) nor BQ-123 (*E*max = 0.35 ± 0.17%, *n* = 9) or BQ-788 (*E*max = 0.21 ± 0.06%, *n* = 9) pretreatment altered the blunted ET-1-induced relaxant response in contralateral carotid rings (one-way ANOVA; Bonferroni post hoc test, *P* < 0.01) (Figure 5).

**Figure 6:**
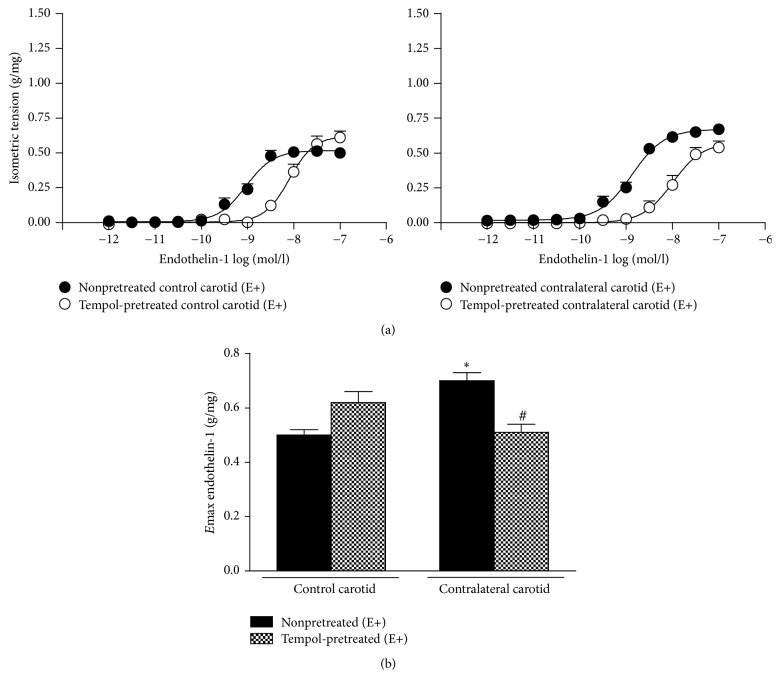
Effect of the SOD mimic Tempol on ET-1-induced contraction in endothelium-intact (E+) contralateral carotid rings removed from operated rats at the sixteenth day after the surgery and in E+ age-matched rats control carotid rings. (a) Cumulative concentration-response curves for ET-1 in carotid rings pretreated or not with Tempol. (b) *E*max values from ET-1-induced contraction in carotid rings pretreated or not with Tempol. Significant difference (*P* < 0.05) from E+ nonpretreated control carotid rings (*∗*) or E+ nonpretreated contralateral carotid rings (#). One-way ANOVA; Bonferroni post hoc test.

Tempol pretreatment did not change ET-1 *E*max values in E+ control carotid rings but restored the endothelinergic contraction in E+ contralateral carotid rings removed from operated rats at the sixteenth day after surgery to the levels obtained in nonpretreated E+ control carotid rings from age-matched rats (Figure 6). ET-1 *E*max in Tempol-pretreated E+ contralateral carotid was not different from the value obtained in this vessel pretreated with Tempol combined with PEG-catalase (0.51 ± 0.23 g/mg, *n* = 9) (one-way ANOVA; Bonferroni post hoc test, *P* < 0.01).

**Figure 7:**
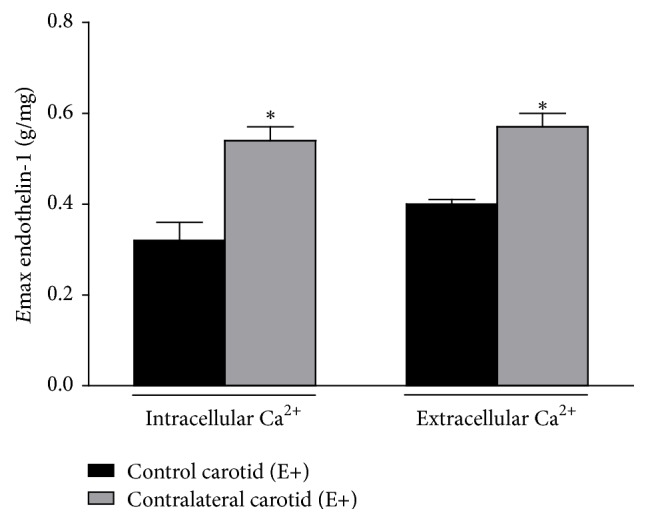
Consequences of balloon injury on ET-1-induced intracellular and extracellular Ca^2+^ mobilization in endothelium-intact (E+) contralateral carotid rings isolated from operated rats at the sixteenth day after surgery. Significant difference (*P* < 0.05) from E+ age-matched rat control carotid rings (*∗*). One-way ANOVA; Bonferroni post hoc test.

*(2) ET-1-Induced Intracellular and Extracellular Ca* ^*2*+^ *Mobilization*. Functional data obtained in assays carried out with Ca^2+^-free solution showed that E+ contralateral carotid rings removed from operated rats at the sixteenth day after surgery exhibited ET-1-induced intracellular Ca^2+^ mobilization values higher than those obtained in E+ age-matched rat control carotid rings. Similarly, Ca^2+^-depleted endothelium-dependent contralateral carotid rings isolated from operated rats at the sixteenth day after surgery, assayed in 1.9 mmol/l Ca^2+^-containing Krebs solution, also exhibited higher values of ET-1-induced Ca^2+^ extracellular mobilization when compared those obtained in E+ age-matched rat control carotid rings, assayed in the same conditions (Figure 7).

**Figure 8:**
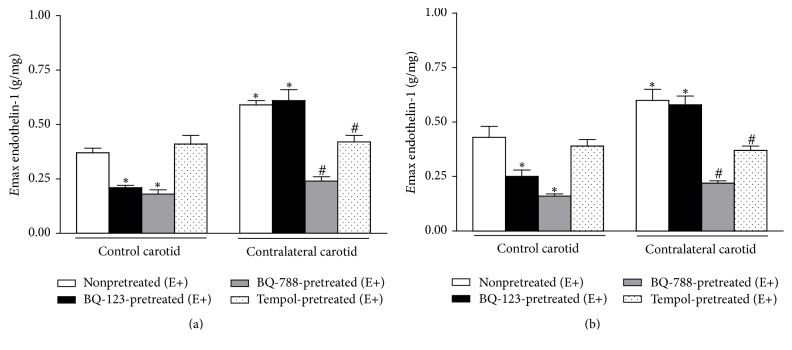
Effect of ET_A_ (BQ-123) or ET_B_ (BQ-788) antagonists or the SOD mimic Tempol on ET-1-induced intracellular (a) and extracellular (b) Ca^2+^ mobilization in endothelium-intact (E+) contralateral carotid rings isolated from operated rats at the sixteenth day after surgery. Significant difference (*P* < 0.01) from rat control carotid rings (*∗*) or rat contralateral carotid rings (#) in the absence of the respective antagonist or scavenger. One-way ANOVA; Bonferroni post hoc test.

ET-1-induced Ca^2+^ intracellular mobilization was significantly reduced by BQ-123 (*E*max = 0.21 ± 0.01 g/mg, *n* = 9) or BQ-788 (*E*max = 0.18 ± 0.02 g/mg, *n* = 9) pretreatments in E+ control carotid rings when compared to the absence of the antagonists. Similarly, ET-1-induced Ca^2+^ extracellular mobilization was significantly reduced by BQ-123 (*E*max = 0.25 ± 0.03 g/mg, *n* = 9) or BQ-788 (*E*max = 0.16 ± 0.01 g/mg, *n* = 9) pretreatments in E+ control carotid rings. In turn, BQ-123 pretreatment did not alter ET-1-induced Ca^2+^ intracellular (*E*max = 0.61 ± 0.05 g/mg, *n* = 9) or extracellular (*E*max = 0.58 ± 0.04 g/mg, *n* = 9) mobilization in E+ contralateral carotid rings removed from operated rats at the sixteenth day after surgery when compared to the absence of the antagonists. However, BQ-788 pretreatment significantly reduced ET-1-induced Ca^2+^ intracellular (*E*max = 0.24 ± 0.02 g/mg, *n* = 9) or extracellular (*E*max = 0.22 ± 0.01 g/mg, *n* = 9) mobilization in E+ contralateral carotid rings removed from operated rats at the sixteenth day after surgery to the levels obtained in E+ age-matched rat control carotid rings pretreated with the respective antagonist (one-way ANOVA; Bonferroni post hoc test, *P* < 0.01) (Figure 8).

Tempol pretreatment did not alter ET-1-induced Ca^2+^ intracellular (*E*max = 0.41 ± 0.04 g/mg, *n* = 9) or extracellular (*E*max = 0.39 ± 0.03 g/mg, *n* = 9) mobilization in E+ control carotid rings when compared to the absence of the antagonists. Nevertheless, Tempol pretreatment restored ET-1-induced Ca^2+^ intracellular (*E*max = 0.42 ± 0.03 g/mg, *n* = 9) or extracellular (*E*max = 0.37 ± 0.02 g/mg, *n* = 9) mobilization in E+ contralateral carotid rings removed from operated rats at the sixteenth day after surgery to the levels obtained in E+ age-matched rat control carotid rings in the presence of the SOD mimic (one-way ANOVA; Bonferroni post hoc test, *P* < 0.01) (Figure 8).

**Figure 9:**
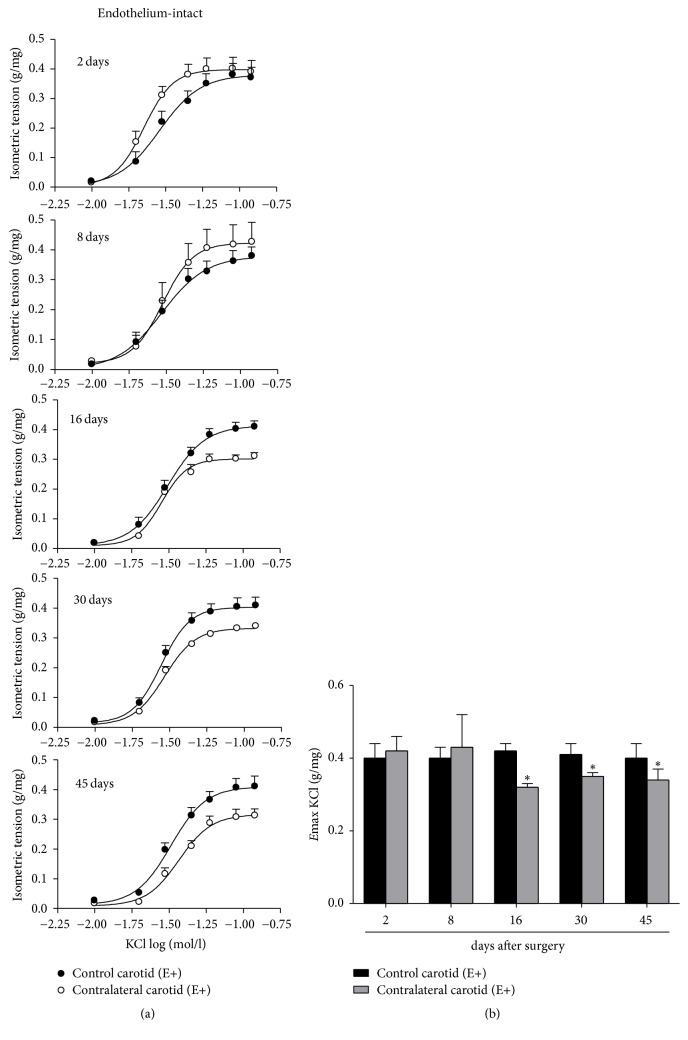
Temporal consequences of balloon injury on depolarization-dependent contraction in contralateral carotid rings isolated from operated rats at 2, 8, 16, 30, or 45 days after the surgery. (a) Cumulative concentration-response curves for ET-1 in endothelium-intact (E+) carotid rings. (b) *E*max values from ET-1-induced contraction in E+ carotid rings. Significant difference (*P* < 0.05) from E+ control carotid rings from the respective age-matched intact rat (*∗*). One-way ANOVA; Bonferroni post hoc test.

*(3) Depolarization-Dependent Contraction*. KCl-induced *E*max values were similarly increased in E+ contralateral carotid rings from the sixteenth day till the forty-fifth day after surgery when compared to the respective E+ control carotid rings isolated from age-matched intact rats (Figure 9).

#### 3.1.3. Chemiluminescence Data

**Figure 10:**
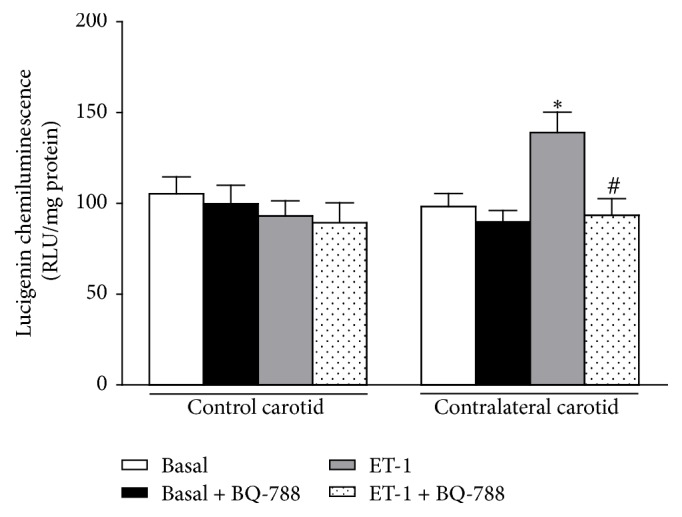
Effect of ET_A_ (BQ-123) or ET_B_ (BQ-788) antagonists on basal or ET-1-induced NADPH oxidase-driven O_2_^−^ production in endothelium-intact (E+) contralateral carotid rings isolated from operated rats at the sixteenth day after surgery. Significant difference (*P* < 0.01) from the respective rat control carotid rings (*∗*) or rat contralateral carotid rings (#). One-way ANOVA; Bonferroni post hoc test.

Basal lucigenin chemiluminescence in contralateral carotid isolated from operated rats at the sixteenth day after surgery (98.25 ± 7.16 RLU/mg protein, *n* = 9) is not different from that one obtained for age-matched rat control carotid (105.34 ± 9.27 RLU/mg protein, *n* = 9). ET-1 significantly increased the basal luminescent signal in contralateral carotid (139.04 ± 11.25 RLU/mg protein, *n* = 9) but not in control carotid (92.99 ± 8.42 RLU/mg protein, *n* = 9). BQ-788 did not alter basal lucigenin chemiluminescence in contralateral carotid (89.72 ± 6.38 RLU/mg protein, *n* = 9) but restored the ET-1-induced signal in this vessel (93.44 ± 9.10 RLU/mg protein, *n* = 9) to the levels obtained in ET-1-stimulated control carotid pretreated with the antagonist (90.03 ± 8.22 RLU/mg protein, *n* = 9) (one-way ANOVA; Bonferroni post hoc test, *P* < 0.001) (Figure 10).

#### 3.1.4. ELISA Data

**Figure 11:**
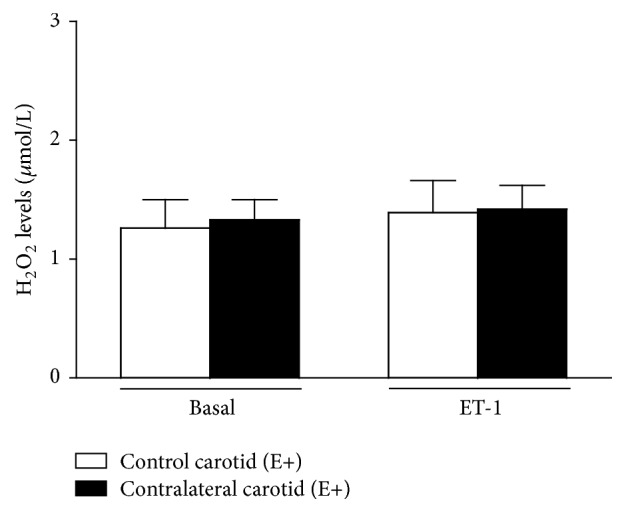
Consequences of balloon injury on basal or ET-1-induced H_2_O_2_ production in endothelium-intact (E+) contralateral carotid rings isolated from operated rats at the sixteenth day after surgery. One-way ANOVA; Bonferroni post hoc test.

Basal (1.33 ± 0.17 *μ*mol/L, *n* = 9) or ET-1-stimulated (1.42 ± 0.20 *μ*mol/L, *n* = 9) H_2_O_2_ levels in contralateral carotid isolated from operated rats at the sixteenth day after surgery were not different from each other or from basal (1.26 ± 0.24 *μ*mol/L, *n* = 9) or ET-1-stimulated (1.39 ± 0.27 *μ*mol/L, *n* = 9) H_2_O_2_ levels in age-matched rat control carotid (one-way ANOVA; Bonferroni post hoc test, *P* > 0.05) (Figure 11).

### 3.2. Discussion

The major new findings from the present study show for the first time that the activation of SP NK_1_ receptors during neurocompensatory response to rat carotid balloon angioplasty triggers a contractile hyperreactivity to ET-1 in contralateral carotid by enhancing the muscular ET_B_-mediated generation of NADPH oxidase-derived O_2_^−^, which upregulates local extracellular and intracellular Ca^2+^ mobilization due to the activation of Ca^2+^ channels other than the voltage-dependent ones at plasma membrane and sarcoplasmic reticulum. Such harmful distant effect assigned to balloon angioplasty consists of a remodeling-independent disorder of contralateral carotid endothelinergic functionality since it was not followed by significant changes in the histological arrangement from the vascular wall.

Our data show that balloon injury triggers the formation of a neointima layer that gradually thickens in the endothelium-denuded ipsilateral carotid from the eighth day till the forty-fifth day after surgery, as previously described [5, 6, 13–16, 35, 36]. The restenotic remodeling that takes place at ipsilateral carotid results from an inflammatory response to balloon rubbing that involves the immediate endothelial denudation and medial disruption, followed by the early intimal proliferation of vascular smooth muscle cells differentiated into the synthetic phenotype [35, 36]. Restenotic ipsilateral carotid exhibits endothelinergic contractile and relaxant hyporresponsiveness since ET-1-induced contraction is drastically reduced while ET-1-induced relaxation is blunted. The general hyporresponsiveness of ipsilateral carotid to vasoactive agents has been correlated to the synthetic phenotype assumed by neointimal smooth muscle cells, which did not contain enough muscle fibers to respond to contractile or relaxant stimuli [5, 6, 12–16].

Our results show that ipsilateral carotid restenosis is not followed by morphological changes in the contralateral artery, whose histological arrangement stays similar to the carotid wall from control (intact) age-matched rats, in agreement with previous findings [5, 6, 11–16, 36]. The unaltered structure from contralateral carotid wall had incorrectly supported the use of this vessel as the control parameter from ipsilateral carotid [36] until the findings provided by Accorsi-Mendonça et al. [5], who described that contralateral carotid exhibits a broad muscular dysfunction resultant from a vascular bed-dependent mechanism not mediated by humoral factors but elicited by the neurocompensatory response to balloon injury [11].

Similar to the angiotensinergic contractile hyperreactivity observed in contralateral carotid at the fifteenth day after balloon angioplasty [5, 16], the contraction induced by ET-1 is markedly increased in this vessel at the sixteenth day after surgery. The endothelinergic contractile hyperreactivity in contralateral carotid compensates ipsilateral hyporresponsiveness, which is typical from neurocompensatory response to balloon injury [5, 6, 12–16]. As previously suggested for angiotensinergic functionality [5, 14], the endothelinergic contractile hyperreactivity of contralateral carotid involves the neurocompensatory response as inductive mechanism since the blockade of NK_1_ receptors restored this response, confirming the role of SP in the functional distant effects of balloon angioplasty. Interestingly, endothelium removal did not increase ET-1-induced contraction in contralateral carotid, in agreement with previous findings obtained for AngII [5, 16]. In agreement with these findings, we also observed that the endothelium-dependent ET_B_-mediated relaxation induced by ET-1, previously characterized as a nitrergic mechanism in rat carotid [26], was completely absent in contralateral carotid at the sixteenth day after surgery, which strongly suggest that neurocompensatory response blunts contralateral endothelial function. Finally, the endothelinergic contractile hyperreactivity of contralateral carotid and the loss of the local endothelial modulation of this response were held until the forty-fifth day after surgery, in close similarity to the previous findings obtained for AngII [5, 16]. Taken together, these findings suggest that the neurocompensatory response to carotid balloon injury enhances the endothelinergic functionality in contralateral carotid by a muscular-dependent mechanism seemingly resultant from the crosstalk with the local angiotensinergic system [20], whose functionality is also upregulated [5, 16]. Accordingly, the crosstalk between ET-1 and AngII involves the upregulation of ET-1 expression upon AT_1_ activation and the subsequent participation of ET-1 as the final mediator of AngII-induced redox and ionic effects [20].

The muscular generation of the contractile factor that positively modulates ET-1-induced contraction in contralateral carotid may be mediated by muscular endothelin receptors. Both endothelin ET_A_ and ET_B_ receptors are highly expressed in vascular smooth muscle cells [37]. As metabotropic receptors coupled to the G_q_ protein, both ET_A_ and ET_B_ receptors mediate ET-1-induced contraction by a Ca^2+^-dependent mechanism [26], which suggest that the contractile factor that contributes to endothelinergic hyperreactivity in contralateral carotid could be generated upon the increase of Ca^2+^ intracellular levels. Among the known contractile factors generated by this kind of signaling pathway underlying the activation of muscular endothelin receptors, NADPH oxidase-derived O_2_^−^ is the most important for increasing arterial tonus during pathophysiological mechanisms typical from restenosis-related diseases such as hypertension and atherosclerosis [37]. In turn, the contractile effects of O_2_^−^ in vascular smooth muscle cells involve the activation of all types of Ca^2+^ channels [38]. Based on these assumptions, we investigated the role of endothelin receptors, NADPH oxidase-derived O_2_^−^, and Ca^2+^ mobilization in the endothelinergic hyperreactivity exhibited by the contralateral carotid. Our findings show that both the ET_B_ antagonist BQ-788 and the SOD mimic Tempol restored ET-1-induced contraction in contralateral carotid, which suggests the ET_B_-mediated muscular generation of O_2_^−^. Indeed, this functional finding that points O_2_^−^ as the final mediator of the contractile endothelinergic hyperreactivity in contralateral carotid is reinforced by the fact that the local ET-1-induced contraction in the presence of Tempol was not altered by PEG-catalase, which excludes the hypothesis that the restoring effect of Tempol could be resultant from relaxant actions triggered by H_2_O_2_ derived from Tempol-induced O_2_^−^ dismutation [12]. Interestingly, ET-1-induced Ca^2+^ extracellular and intracellular mobilizations were increased while the depolarization-dependent contraction induced by KCl was reduced in contralateral carotid, suggesting that the endothelinergic hyperreactivity results from the activation of Ca^2+^ channels other than the voltage-dependent ones at plasma membrane and sarcoplasmic reticulum. Accordingly, the functional hypothesis of the positive modulation played by ET_B_-derived O_2_^−^ on ET-1-induced activation of Ca^2+^ channels in contralateral carotid was confirmed by the restoring effects of BQ-788 and Tempol on ET-1-induced Ca^2+^ mobilization and the inhibitory effects of BQ-788 on ET-1-induced increase in the basal NADPH oxidase-driven O_2_^−^ generation in this vessel. Added to these findings, the unaltered ET-1-induced levels of H_2_O_2_ in contralateral carotid reinforce the conclusion that O_2_^−^ but not H_2_O_2_ mediates Ca^2+^ channels activation during ET-1 stimulus in this vessel. By the way, the unaltered ET-1-induced levels of H_2_O_2_ in contralateral carotid point an enough conversion of all H_2_O_2_ quantum derived from both the dismutation of ET_B_/NADPH oxidase-induced O_2_^−^ and the eventual Tempol-induced O_2_^−^ dismutation, which may be related to a putative increase in local catalase and/or peroxiredoxins activity and/or expression. This hypothesis would also explain the ineffectiveness of PEG-catalase on the restoring effects of Tempol in contralateral carotid.

The loss of the negative modulation played by ET_A_ and ET_B_ receptors on ET-1 induced contraction in contralateral carotid, as suggested by the ineffectiveness of BQ-123 or BQ-788 in increasing this response as well as by the local blunted ET-1-induced relaxant response, clearly points a local endothelial dysfunction extended to endothelinergic relaxation mechanisms. Indeed, endothelial ET_B_ receptors mediate a relaxant response induced by ET-1 upon a nitrergic signaling [26], whose functionality is impaired by the oxidative stress in contralateral carotid [5, 6, 11–16]. This endothelial endothelinergic dysfunction contributes to the contractile effects of the muscular ET_B_-derived O_2_^−^ for enhancing the endothelinergic functionality in contralateral carotid.

## 4. Conclusions

In summary, our study describes for the first time that the activation of SP NK_1_ receptors during the neurocompensatory response to carotid balloon injury enhances the endothelinergic functionality of rat contralateral carotid. The mechanism underlying the contractile hyperreactivity of contralateral carotid to ET-1 involves the upregulation of ET-1-induced Ca^2+^ mobilization due to the activation of Ca^2+^ channels nonvoltage-dependent by muscular ET_B_-mediated NADPH oxidase-derived O_2_^−^. The pathophysiological significance of endothelin system to the distant harmful effects of balloon injury opens a new perspective for the development of effective therapeutic approaches to prevent postangioplasty complications.

## Supplementary Material

Endothelin-1 (ET-1)-induced maximum contraction (*E*max) values in endothelium-intact ipsilateral (left) or contralateral (right) carotid rings from sham-operated rats (8, 16, 30 or 45 days after surgery) compared to endothlium-intact carotid rings isolated from age-matched intact control rats.
