# Supplementary figures and images for: Endothelinergic Contractile Hyperreactivity in Rat Contralateral Carotid to Balloon Injury: Integrated Role for ETB Receptors and Superoxide Anion
Source: Biomed Res Int. 2017 Sep 14;2017:3137580. doi: 10.1155/2017/3137580 (PMC5618786; doi:10.1155/2017/3137580)

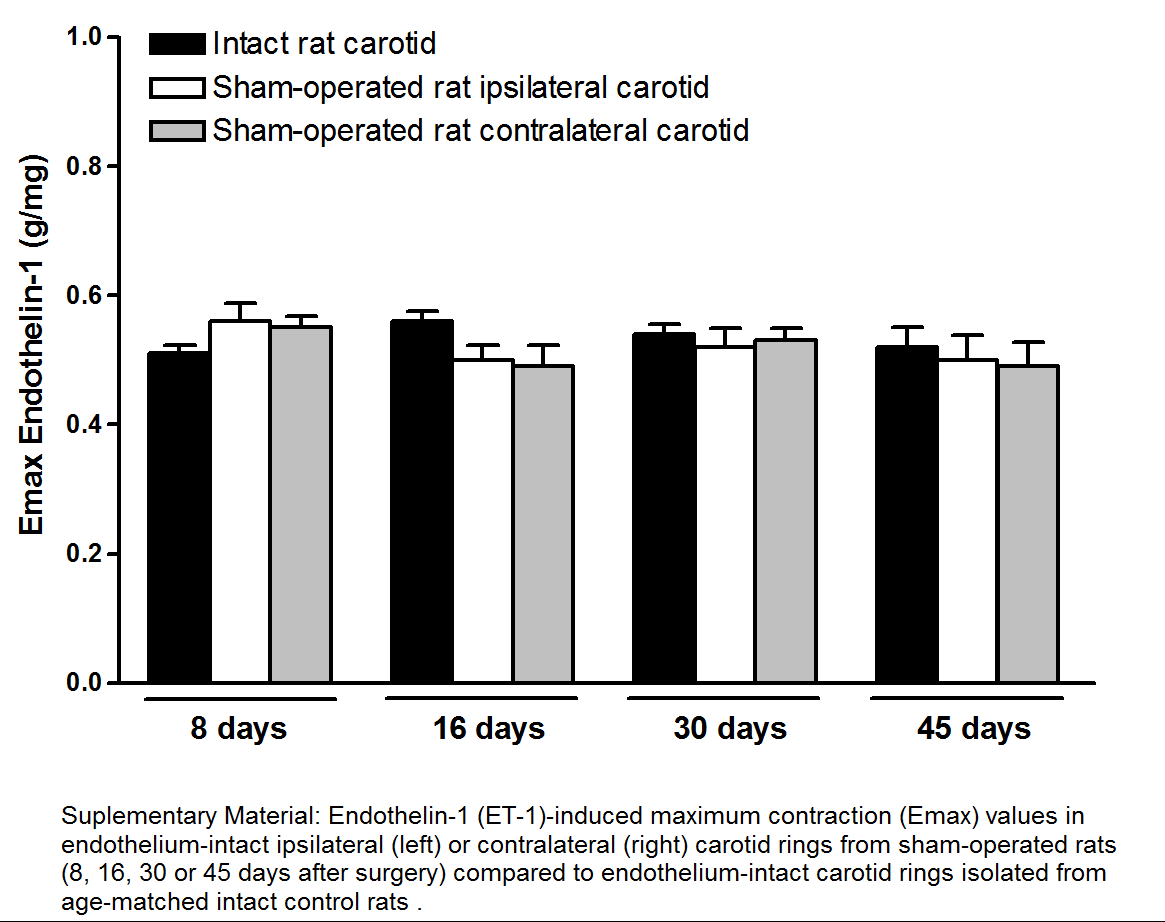

Supplement: Supplementary file 1 — Endothelin-1 (ET-1)-induced maximum contraction (Emax) values in endothelium-intact ipsilateral (left) or contralateral (right) carotid rings from sham-operated rats (8, 16, 30 or 45 days after surgery) compared to endothlium-intact carotid rings isolated from age-matched intact control rats. [file 3137580.f1.tif]
